# A diffusion model conditioned on compound bioactivity profiles for generating high-content images

**DOI:** 10.1038/s41598-026-44976-6

**Published:** 2026-04-03

**Authors:** Steven Cook, Jason Chyba, Laura Gresoro, Doug Quackenbush, Minhua Qiu, Peter Kutchukian, Eric J. Martin, Peter Skewes-Cox, William J. Godinez

**Affiliations:** 1 Novartis Biomedical Research, San Diego, 92121 CA USA; 2 Novartis Biomedical Research, Cambridge, 02139 MA USA; 3 Novartis Biomedical Research, Emeryville, 94608 CA USA

**Keywords:** Generative AI for drug discovery, In silico HCI, Virtual screening, Phenotypic screening, Virtual screening, Machine learning, Virtual drug screening

## Abstract

High-content imaging (HCI) provides a rich snapshot of compound-induced phenotypic outcomes that augment our understanding of how compounds affect cellular systems. Generative imaging models for HCI provide a route towards anticipating the phenotypic outcomes of chemical perturbations in silico at unprecedented scale and speed. Here, we developed Profile-Diffusion (pDIFF), a generative method leveraging a profile-to-image latent diffusion model conditioned on in silico bioactivity profiles to generate high-content images displaying the cellular outcomes induced by compound treatment. We trained and evaluated a pDIFF model using high-content images from a Cell Painting assay profiling 3750 molecules (3375 training compounds and 375 held-out compounds) with corresponding in silico bioactivity profiles. Using the held-out set we demonstrate that pDIFF provides improved visual depictions of phenotypic responses of compounds that are structurally dissimilar to training compounds, compared to a baseline profile-to-image latent diffusion model trained on substructural molecular descriptors only. In a virtual hit expansion scenario, pDIFF yielded statistically significant improvement in expansion outcomes as measured by nearest-neighbor retrieval accuracy, compared to expansions based on compound structural representations, bioactivity profiles, and generative imaging models based only on substructural molecular descriptors, thus showcasing the potential of the methodology to speed up and improve the search for novel phenotypically active molecules.

## Introduction

Compound-induced phenotypes observed through high-content imaging (HCI) assays provide rich clues as to the activity and mechanisms of compounds in cellular systems^1^. While HCI assays can screen large compound collections, experimental assays are still restricted to a comparatively small sample of the synthetically accessible chemical space^2–4^. Generative models that predict images containing the specific phenotype induced by any compound can enable virtual screening and profiling of compound collections at unprecedented scale, speed, and cost^5^.

Early work in generative models for cell microscopy images focused on mapping a symbolic representation of cellular biology (e.g., a state vector) onto an image using explicit appearance models for image generation^6–8^. More recent approaches leverage advances in deep neural networks^9^ to learn image generation functions directly from training images^10–12^. Conditioning the generation functions with compound representations, such as compound fingerprints, allows these methods to generate high-content images showing the phenotypic outcomes induced by compound treatment^5,13^. Architecturally, these methods are built upon flow-based generative models^5^ and generative adversarial networks (GANs)^13^. Recent research points to the superior efficacy of diffusion−based generative architectures for conditional image generation^14^, and more broadly, for drug discovery tasks^15,16^. Chemically, these methods are conditioned on compound representations derived directly from molecular structures, limiting model generalizability to structurally similar chemical matter^5,13^. Alternative representations based on imputed compound bioactivity^17^ can potentially improve the ability of such generative methods to extrapolate to novel chemical matter.

In this paper, we introduce Profile Diffusion (pDIFF), an approach combining a stable diffusion-based generative model with in silico bioactivity profiles to generate high-content images displaying the cellular outcomes induced by compound treatment. Generating high-content images instead of predicting image features allows for flexible downstream application as any desired features can then be computed separately on the pDIFF output images. pDIFF model training only requires pairs of vectorial compound representations and associated high-content images, without requiring *a priori knowledge of the distribution of signal intensities or morphological features.* We build and validate a pDIFF model with a collection of bioactivity profiles and Cell Painting images corresponding to a diverse chemogenomic library of 3750 compounds^18^ (see Supplementary Figure 1 for calculated molecular properties). Using a “realistically novel” split for validation^17^, we demonstrate that pDIFF improved extrapolation to novel chemical matter compared to a baseline diffusion model conditioned on chemical fingerprints. We also test the pDIFF model in a virtual hit expansion scenario and show that pDIFF yields statistically significant improvement in hit expansion outcomes.

## Results

### Profile diffusion: stable diffusion model conditioned on compound profiles

**Fig. 1:**
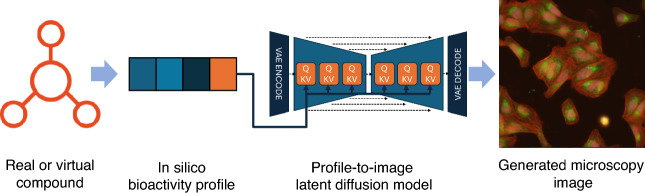
Profile-Diffusion (pDIFF) workflow. A virtual or real compound is represented computationally through an in silico bioactivity profile computed by pQSAR. The representation is used to condition the generative process of the stable diffusion model underlying pDIFF to generate a high-content image showing the phenotypic outcome induced by compound treatment.

We developed the Profile Diffusion (pDIFF) methodology based off the stable diffusion architecture^19^. At training time, pDIFF takes as input an in silico bioactivity profile as well as a high-content image displaying the cellular outcomes induced by that compound. The input image is compressed into a lower-resolution latent representation through a pre-trained variational autoencoder (VAE)^20^. A latent diffusion network conditioned on the compound’s bioactivity profile is trained in the VAE’s latent space to predict the noise added to the latent image at each step of a diffusion process (see Fig. 1 and Methods). Thus, in comparison with the original implementation of the Stable Diffusion model, where natural language embeddings are used to condition the denoising network, pDIFF instead induces the model to learn the “language of compound bioactivity”.

To generate an image for a specific compound with a trained pDIFF model, we take the in silico bioactivity profile of that compound, sample a random noise tensor in the VAE’s latent space, and iteratively denoise that tensor with the model conditioned on the compound’s bioactivity profile together with a denoising diffusion probabilistic models (DDPM) schedule^21^ (see Methods). Varying the number of denoising iterations trades off image quality against computation time, as the model must be invoked more often for more steps. Finally, the denoised tensor is passed through the VAE decoder to synthesize a high-content image. Different random noise tensors result in different images, thus allowing generation of a collection of images for each input compound.

### Profile diffusion generalizes to novel chemical matter

To validate pDIFF in a realistic drug discovery context, we used 3750 compounds of the Novartis Mechanism-of-Action (MoA) Box chemogenomic library (MoA Box)^18^. These compounds were profiled in a Cell Painting assay^1^, where U-2 OS cells were compound-treated in triplicate at 12.5 $\mu$M (see Methods). Twenty-four high-content images were acquired per compound. We utilize the cytoplasm, mitochondria, and nuclei channels of the Cell Painting protocol to construct three-channel RGB images that, along with their corresponding compound profiles, form the dataset for model building and evaluation.

As conditioning profile for pDIFF, we used the in silico bioactivity profiles predicted by Profile-QSAR (pQSAR)^17^, a massively multitask bioactivity machine learning model trained on over two million compounds across 14222 Novartis dose-response assays (see Methods). For each of the 3750 compounds in our dataset, pQSAR predicts activity in terms of $\text {pAC}_{50}$ values (which is the negative logarithm of the half-maximal activity concentration) for each of the 14222 assays, thus resulting in a 14222-length profile per compound. For numerical tractability, we reduced predictions from the 14222 assays to 2018 assays through a target-focused assay selection scheme (see Methods).

For benchmarking purposes we also trained a stable diffusion model conditioned on chemical fingerprints. Specifically, we used extended-connectivity fingerprints (ECFPs)^22^, each folded into a 2048 count vector. Briefly, ECFPs indicate the presence or absence of substructural compound motifs defined using hashing algorithms, permitting intersection-over-union similarity measures such as Tanimoto similarity. For this baseline model, we used the same training and inference regimes as used for pDIFF. At inference time, we set both pDIFF and the baseline model to generate 12 images per compound (see **Methods**).

To validate the performance of pDIFF and the baseline model, we used a “realistically novel” cluster-based split approach^17^. This approach aims to replicate a realistic screening scenario, where project teams are interested in testing compounds that differ significantly from those previously tested (see **Methods**). We chose 10% of the total dataset size for the held-out set, giving us 3375 training and 375 held-out compounds. This split results in a highly dissimilar median Tanimoto coefficient of 0.11 between train and test compounds (Supplementary Figure 2).

**Fig. 2:**
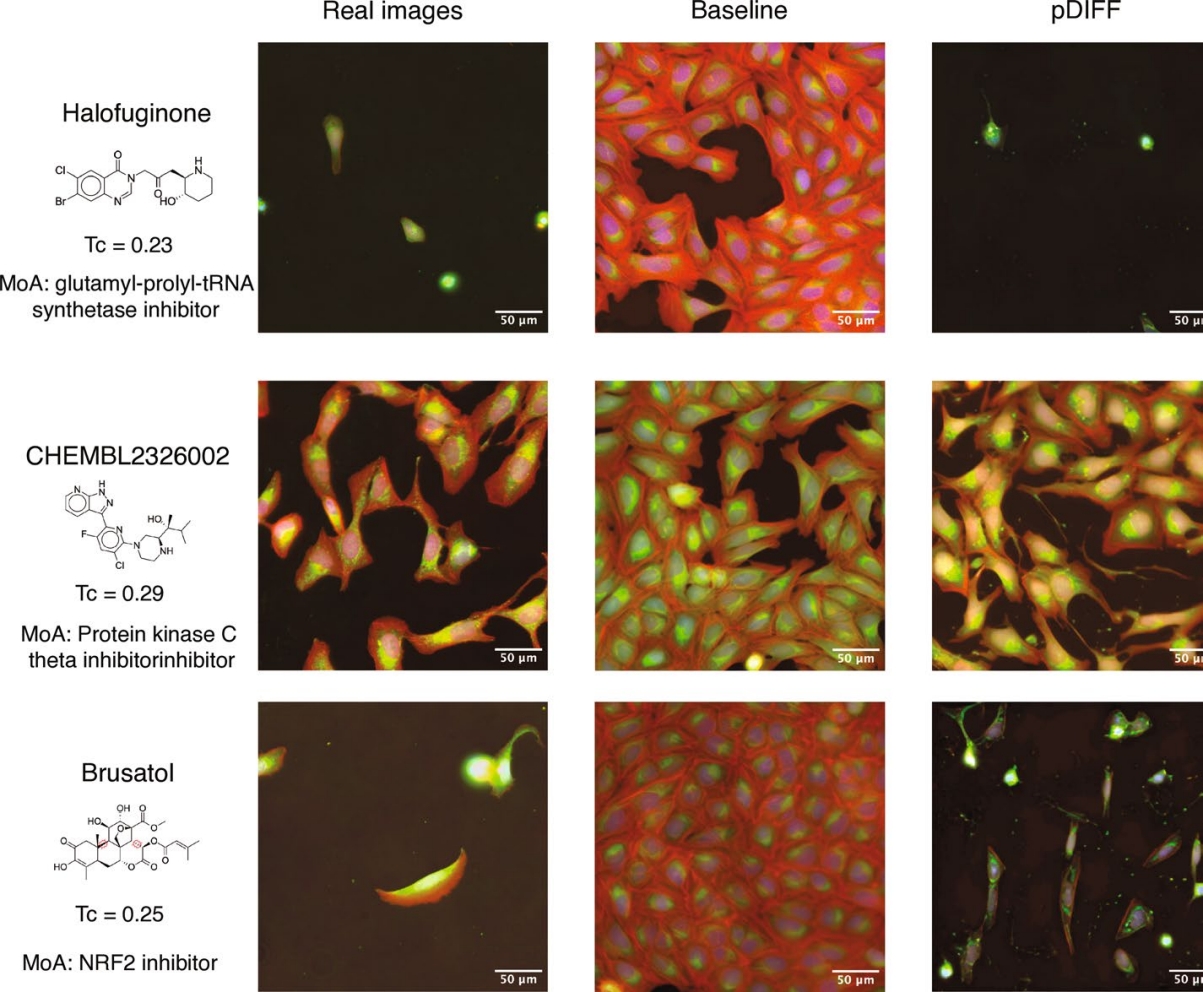
Molecules in the realistic held-out set along with their corresponding real images (Nuclei, blue; mitochondria, green; red, cytoplasm) as well as images generated with diffusion models. We show images generated by a baseline diffusion model conditioned on chemical fingerprints (Baseline) as well as by pDIFF. For each molecule, we list the Tanimoto coefficient (Tc) to the nearest neighbor molecule in the training set as well as the associated mechanism of action (MoA).

Example real and generated images for three held-out compounds are shown in Fig. 2. These active compounds induce outcomes visually different from those observed in the neutral controls (see Supplementary Figure 3). We show images for Halofuginone, a glutamyl-prolyl-tRNA synthetase inhibitor^23^ that is known to inhibit the viability of Osteosarcoma cell lines^24^. The baseline model conditioned on chemical fingerprints is unable to correctly predict the cell death outcome induced by this compound, which has a low chemical similarity to the training set (Tanimoto coefficient of 0.23 to the nearest neighbor compound in the training set). In contrast, the pDIFF model, which is conditioned on pQSAR bioactivity profiles, visually recapitulates this outcome. We also show images for CHEMBL2326002, a protein kinase C-theta inhibitor^25^ which induces a slightly elongated phenotype with a punctuated pattern in the mitochondria channel. The baseline model does not predict the elongated punctuated pattern, whereas pDIFF generates images with cells closely resembling the phenotype induced by this compound. For Brusatol, which plays a role in DNA damage repair^26^ by acting as an inhibitor of the NRF2 pathway^27^, cells exhibit a toxic phenotype that is anticipated only by pDIFF.

Additional images for molecules are shown in **Supplementary Figure** 4. Across a variety of molecules, mechanisms of action (viz. actin polymerization, LATS inhibition, antifungal activity, v-ATPase inhibition, viral fusion inhibition, HSP90 inhibition, BRD4 inhibition), and phenotypic responses (e.g., punctae, elongation, filamentation, clumping), pDIFF generated images displaying phenotypes that closely resembled those observed in the real images. For Thailandepsin A^28^, an HDAC inhibitor, pDIFF generated images displaying elongated cells while the real images display a phenotype more similar to the neutral controls. As cell lines vary in their expression of cellular targets and pathways^29^, we hypothesize that pDIFF, learning from other HDAC inhibitors in the training set, predicted an outcome not induced in the U2-OS cell line used in this study. We also show images for pterocarpanquinone, which modulates FoxO3a activity^30^. For this molecule both the baseline diffusion model and the pDIFF model did not recapitulate the apoptotic phenotype.

To quantitatively assess the ability of pDIFF to recapitulate phenotypic outcomes, we follow a previous scheme^5^ by computing biologically relevant features (viz. coverage, cell count, cell size) for real and generated image sets. To calculate these segmentation-dependent features, we merge the channels to create grayscale images, then utilize the Cellpose^31^ segmentation model to create cell masks from which image-level cell coverage, count, and average cell size metrics can be directly computed. Features are averaged over all images for each compound, then Spearman correlation coefficients between these compound-aggregated features of real and generated image sets are calculated (see Methods).

Table 1 shows the real vs. generated correlation coefficients across the 375 held-out (test) compounds for three features. As upper bound, we compute the correlation among real images of the held-out compounds by splitting the 24 real images into two halves, calculating features for each half, and computing the correlation coefficients between the features of both halves. Correlation values for the image features between the two sets of real images range from 0.48 to 0.67. For this challenging held-out set of compounds with low chemical similarity to the training set (median Tanimoto coefficient of 0.11 between train and test compounds, cf. Supplementary Figure 2.) , the baseline diffusion model conditioned on chemical fingerprints yielded correlation values ranging from 0.04 to 0.13. The pDIFF model yielded correlation coefficients between 0.12 to 0.48, thus exhibiting substantially improved performance on novel chemical matter compared to the baseline model.

**Table 1:** Results for held-out validation compounds. Values given are Spearman correlation coefficients between compound-aggregated average values for each image feature. The first row shows the correlation values for each half of the real images compared against each other. pDIFF model performs better than the baseline diffusion model conditioned on chemical fingerprints.

	Coverage	Cell Count	Cell Size
Real images upper bound	.67	.56	.48
Baseline diffusion model	.13	.10	.04
pDIFF	.48	.28	.12

### Profile diffusion enables improved retrieval of phenotypically similar molecules for hit expansion

Having ascertained the ability of pDIFF to generalize to novel chemical matter, we proceeded to test pDIFF in a virtual hit expansion scenario, where the goal is to find compounds inducing phenotypic outcomes similar to those induced by known active compounds (i.e., phenotypic screening hits). To set the ground truth for this scenario, we take the real images of 101 active query compounds from the training set and compare these to the real images of the 375 compounds in the held-out (test) set. To compare the similarity of two compounds via images, we use the Sinkhorn divergence^32^ on the sets of Cellpose^31^ feature vectors derived from the compounds’ corresponding images. Using these Sinkhorn divergences, we retrieve the 50 nearest-neighbor compounds in the test set for each query compound in the training set. We repeat the nearest neighbor retrieval but with pDIFF-generated images instead of real images for the test compounds, thus comparing real images for query compounds with pDIFF synthetic images for test compounds. As performance measure for this task, we calculate the percentage overlap between the ground truth nearest neighbors and those retrieved using pDIFF images.

**Fig. 3:**
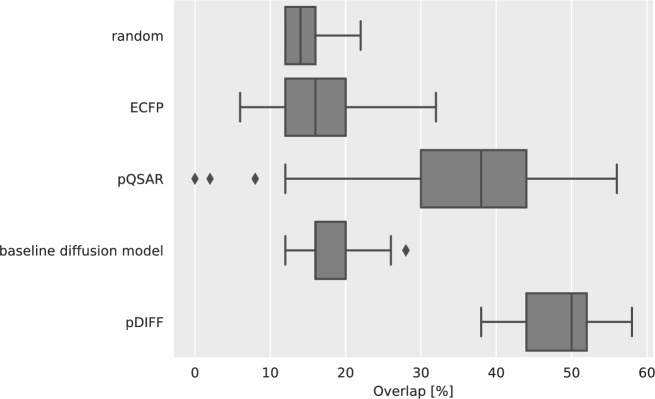
Distribution of percentage overlap values between 50 ground truth nearest neighbors and those retrieved with virtual expansion approaches for 101 query compounds. The query compounds are actives from the training set, and we query into the 375 compounds of the held-out set. We show the performance for an approach selecting molecules randomly (‘random’) as well as for similarities computed using chemical or bioactivity profiles (ECFP and pQSAR, respectively). The results for approaches computing similarities using image profiles derived from images generated through either a baseline diffusion model or pDIFF are also shown.

We report the distribution of percentage overlap between the real and pDIFF test-set nearest-neighbors for the 101 query compounds in Fig. 3. As baseline, we show the percentage overlap values for 101 random selections of 50 test compounds, which yields a median percentage overlap of 14%. As additional baselines, we show the percentage overlap values for nearest neighbors retrieved with chemical fingerprints as well as bioactivity profiles (ECFP and pQSAR profiles, respectively) using a Tanimoto or cosine distance. These approaches lead to median percentage overlaps of 16% and 38%, respectively. A baseline diffusion model conditioned on ECFPs leads to a median percentage overlap of 16%. Finally, retrieval with images generated by pDIFF leads to a median percentage overlap of 50%. A two-sample Kolmogorov–Smirnov test^33^ as implemented in the SciPy library^34^ was used to determine the statistical significance of differences among the percentage overlap values of the different approaches. The resulting p-values were corrected for false discovery rate with the Benjamini–Hochberg method (see Supplementary Table 1). The differences between the distribution of percentage overlap values of the pDIFF approach and those of all other approaches were significant. This outcome suggests that pDIFF enables improved retrieval of phenotypically similar molecules, thus enhancing virtual expansion outcomes.

## Discussion

High-content screening assays provide rich mechanistic and holistic information about cellular responses to chemical perturbations. Overcoming fundamental limitations in screening capacity and compound synthesis via in silico generated high-content images holds potential to vastly expand the compound search space. To this end, we developed a purely virtual generative imaging approach, pDIFF, necessitating only in silico bioactivity profiles as input for image generation. Using a realistic validation scheme, we show that pDIFF provides remarkably improved image predictions for novel chemical matter.

Generating HCI images instead of predicting image measurements directly from compound representation benefits from state-of-the-art diffusion models that both excel at generating high-quality, high-dimensional images as well as exhibiting more stable convergence^35^. Relevant image-derived measurements reflective of phenotype can subsequently be extracted from the generated images with existing image analysis pipelines. In our study, for example, we use the same analysis pipeline to segment and extract image features for both real and pDIFF-generated images. While the image features could be predicted directly from in silico compound profiles, the ability to generate images is key to gaining the confidence of experimentalists as the generated images readily provide visual insight informing the image-derived measurements. pDIFF allows for visual review of HCI-based virtual screens just as chemists visually review virtual screens from chemical similarity or docking.

In our study, we used the bioactivity profiles calculated by the in-house Profile-QSAR (pQSAR) model^17^ trained on over 2M compounds and 14k Novartis internal dose-response assays. The pQSAR methodology is publicly available, and useful models have been built on public bioactivity databases, such as ChEMBL^36^. pDIFF could also be trained on other experimental profiles such as gene expression signatures^37–39^. Furthermore, augmenting the bioactivity profiles with cell type and concentration information with corresponding image data could expand the predictive scope of a single pDIFF model.

Given its ability to predict phenotypic outcomes for novel chemical matter, we envision pDIFF having impact in a number of drug discovery scenarios. As shown by our findings, hit expansion campaigns searching for molecules inducing phenotypic outcomes similar to those induced by hit molecules can benefit from the improved image-based similarity calculations enabled by pDIFF. Profiling activities^40^ aiming to reveal the mechanism-of-action of molecules can likewise leverage pDIFF generated images for similarity calculations relative to images of a reference set of annotated molecules. Image-based readouts have been shown to be predictive of cardiotoxocity^41,42^ and we anticipate pDIFF-generated images to help flag adverse cardiac effects of compounds. Historical imaging assays could be revisited virtually through pDIFF, so past image screening investments can be leveraged to enable future follow up work.

Training pDIFF is computationally demanding due to the need to re-learn the space of high-content images and their corresponding profiles instead of the CLIP text embeddings Stable Diffusion was trained with. Our training set of 12 fields of view (FOVs) per each of 3,375 compounds, took 360 GPU-hours to train for 30,000 steps. A trained pDIFF model can predict one 512$\times$512 pixel output patch per GPU-second using a conservative, high-quality inferencing schedule (DDPM^21^, 100 network inference steps). Alternative schedulers such as the Diffusion Probabilistic Models (DPM) solver^43^ purport to give a similar quality of results using only tens of steps, making this an attractive area for further optimization. Diffusion model inference is more expensive than other generative models like GANs due to the need to repeatedly invoke the network. Even with our conservative settings, the entire MoA box imageset of 90,000 images (3,750 compounds $\times$ 24 FOVs) could be re-generated via inference in 25 GPU-hours, without need for expensive and time-consuming compound synthesis, plating, image acquisition, etc.

In conclusion, our work shows the potential of in silico image prediction to anticipate induced phenotypic outcomes of compounds, including those generated through other machine learning methods^44–46^, much earlier in the drug discovery pipeline. The application of pDIFF allows us to explore broader and more diverse compound collections at unprecedented speed, scale, and efficiency.

## Methods

### Image acquisition and preparation

U2-OS cells were treated with 3750 compounds from the Novartis MoA Box^18^ at a fixed concentration of 12.5µM and incubated for 24 hours. The Cell Painting staining protocol^1^ was applied. Image acquisition was performed using a 4 HP laser, 4 camera Phenix imaging system and a 20x NA1.0 water immersion objective. Acquired images were background corrected using the BaSIC algorithm^47^, rescaled by a factor of ½ from 2160x2160 pixels to 1080x1080, then random crops of 512x512 are extracted from each image for training. RGB images were assembled from the F-actin Cytoskeleton, Mitochondria, and Nucleus channels (Phalloidin/Alexa, MitoTracker Deep Red, and Hoescht 33342 stainings, respectively).

### Profile calculations

To compute the in silico bioactivity profiles for each compound, we used 14222 models from the massively-multitask pQSAR algorithm for predicting $\text {pAC}_{50}$ values for 14222 Novartis-internal biochemical and cellular dose-response assays. We narrowed the resulting 14222-D bioactivity profile by first selecting only the assays where pQSAR models provided useful predictions on the challenging “realistically novel” held-out set per assay, as measured by the squared Pearson correlation coefficient $r^2> 0.3$ between predicted and experimental $\text {pAC}_{50}$ values. We then selected the target-based biochemical and cellular assays in the narrowed assay collection, grouped them by target protein, and selected the best-performing pQSAR model based on the models’ $r^2$ values per target. For the remaining purely-phenotypic assays, we grouped them by drug discovery project, and likewise selected the best-performing pQSAR model per project. For the remaining set of assays with neither target nor project annotations, we selected only very high-quality pQSAR models ($r^2> 0.9$). This selection process resulted 2018 assays, amounting to a 2018-D in silico bioactivity profile per compound. A block diagram describing this selection process is shown in **Supplementary Figure** 5. The profile was zero-padded i.e., 30 zeros appended to the vector to reach the 2048-D input length required for the pDIFF model.

### Model architecture

The Stable Diffusion 2.1 model^19^ was used as the backbone for pDIFF. The lengths of the cross-attention weights are changed to 2048, and all existing weights are re-initialized. As no natural language inputs are needed, the CLIP module is removed. The pre-trained VAE is retained and its weights are frozen. Following^19^, we train the model to minimize the mean squared error between actual noise $\epsilon$ and predicted noise $\epsilon _\theta (z_t,t,y)$ (Eq. 1), where *t* is a current timestep of the noise schedule, $\theta$ are the network parameters, $z_t$ is the latent image representation at step *t* of the noise schedule, $\mathcal {E}$ is the VAE used to encode the image *x*, and *c* is the conditioning profile.

1 $$\begin{aligned} L = \mathbb {E}_{\mathcal {E}(x),c,\epsilon \sim \mathcal {N}(0,1),t} \biggl [ ||\epsilon - \epsilon _\theta (z_t,t,c)||^2_2 \biggr ] \end{aligned}$$

### Model training

pDIFF was trained for 30k steps on 4x Nvidia A100 GPUs using huggingface Accelerate, no mixed precision, in distributed data parallel mode. Starting from 24 images per compound in the training set, pDIFF is trained using 12 of those images, while the 12 additional images per compound in the training set are held out from training and reserved for calculating an upper bound for expected image similarity. Training time was approximately 90 wall-clock hours (360 GPU hours) for the 40k images in the training set (12 images per compound x 3375 compounds). The Min-SNR weighting strategy^48^ was applied to accelerate convergence, and offset noise^49^ was applied to help the model learn to generate empty and nearly-empty FOV images with very low average intensity. Training hyperparameters are presented in Supplementary Table 2.

### Inference

The DDPM scheduler^21^ with 100 steps was used for inference with the model. 12 images were generated per compound profile, at a time cost of approximately 1 second per 512x512 output image per GPU. We use the guidance algorithm^50^ to drive the generation process. In Eq. 2*w* is the guidance scale, *c* is the conditioning profile, $z_t$ is the output result of the previous denoising step, $\emptyset$ is a null conditioning profile, $\epsilon _t(z_t, \emptyset )$ is the model’s unconditional noise prediction, $\epsilon _t(z_t, c)$ is the conditional noise prediction, and $\tilde{\epsilon _t}$ is the resulting guided noise^50^.

2 $$\begin{aligned} \tilde{\epsilon _t} = (1+w)\epsilon _t(z_t, c) - w\epsilon _t(z_t, \emptyset ) \end{aligned}$$

Inference-time classifier-free guidance scale value $w=4$ was chosen empirically.

### Model validation

We used the “realistically novel” split^17^ to evaluate pDIFF. In this approach, the compounds are clustered by Tanimoto similarity, and the clusters ranked in order of size. We allocated compounds from the larger clusters to the training set until 3375 (90%) of the compounds were included. The remaining 375 (10%) of compounds coming from the singletons and smaller clusters were allocated to the realistic held-out set.

To quantify model performance, we first segmented real and generated images using Cellpose with the default cyto2 model in grayscale mode^31^. Following a previous approach^5^, hand-engineered features were calculated for each image. Specifically, we computed the total image area covered by segmented cells (coverage), the number of segmented cells in the image (cell count), and the size of the segmented cells. We averaged the values of these features over all images corresponding to the same compound and calculated Spearman correlations coefficients between features derived from real and generated images across the 375 held-out, or test, compounds.

### Reproducing the training set

We check that models learned to reproduce phenotypic outcomes by evaluating the performance on the training set. Supplementary Table 3 shows the Spearman correlation coefficients comparing pDIFF generated images for the 3375 molecules in the training set to the real images. As upper bound, we report the correlations of coverage, count, and size metrics between the 12 real images per. Resulting correlation values for the coverage, count, and size metrics in this real-vs-real comparison for the training set compounds range from 0.44 to 0.62. A baseline diffusion model conditioned on chemical fingerprints yields correlation values between 0.15 to 0.41. The pDIFF model shows very realistic performance with correlation values ranging from 0.40 to 0.59.

## Supplementary Information

Supplementary Information.

## Data Availability

The data used in this study are proprietary to Novartis. The data are not publicly available due to intellectual property restrictions. An example dataset is available in the pDIFF code respository.

## Code availability

The code and an example dataset for pDIFF is available in Supplementary Code and at https://github.com/Novartis/pDIFF.
