# Supplementary material for: A diffusion model conditioned on compound bioactivity profiles for generating high-content images
Source: Sci Rep. 2026 Apr 3;16:16032. doi: 10.1038/s41598-026-44976-6 (PMC13199470; doi:10.1038/s41598-026-44976-6)
Supplement: Supplementary file 1 — Supplementary Information. [file 41598_2026_44976_MOESM1_ESM.pdf]

A diffusion model conditioned on compound bioactivity profiles for predicting high-content images

Steven Cook[1*], Jason Chyba[1], Laura Gresoro[1], Doug Quackenbush[1], Minhua Qiu[1], Peter Kutchukian[2], Eric J. Martin[3], Peter Skewes-Cox[3], William J. Godinez[3*]

[1]Novartis Biomedical Research, San Diego, 92121, CA, USA.
[2]Novartis Biomedical Research, Cambridge, 02139, MA, USA.
[3]Novartis Biomedical Research, Emeryville, 94608, CA, USA.

*Corresponding author(s). E-mail(s): steven-1.cook@novartis.com; william_jose.godinez_navarro@novartis.com;

Supplementary Figures

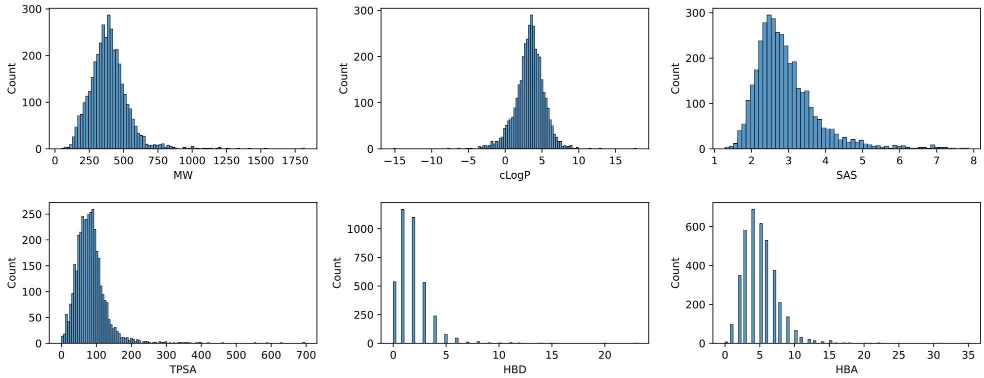

Supplementary Figure 1 Distribution of calculated properties of the 3750 molecules used in this study. Histograms are shown for the following properties: molecular weight, calculated logarithm of the octanol-water partition coefficient (cLogP), synthetic accessibility score (SAS), topological polar surface area (TPSA), number of hydrogen bond donors (HBD), number of hydrogen bond acceptors (HBA).

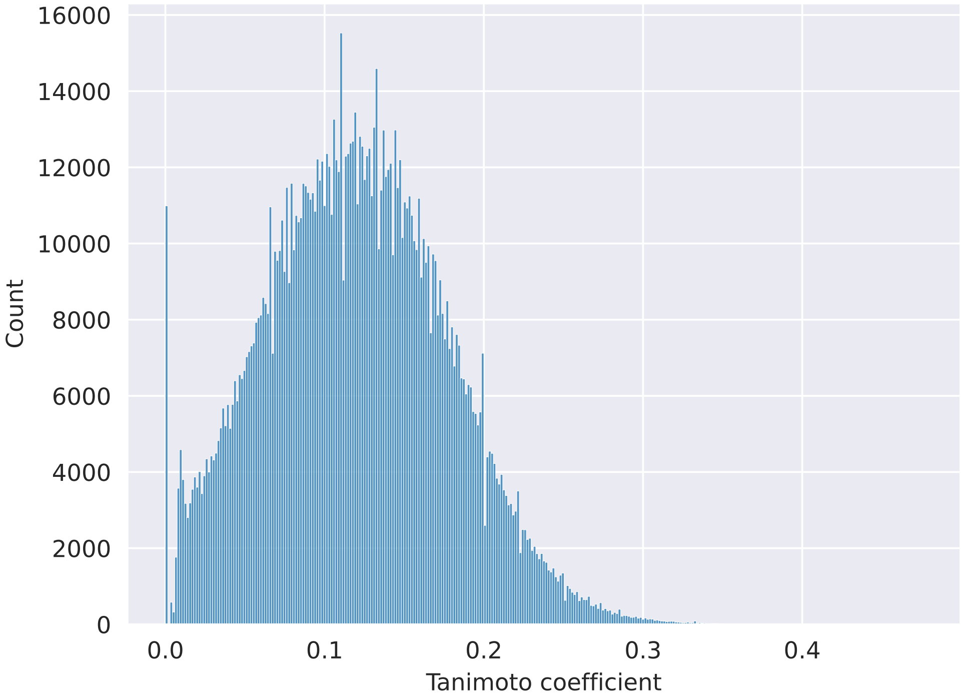

Supplementary Figure 2 Histogram of Tanimoto coefficients between compounds in the training and held-out set for a total of 1265625 comparisons. The median Tanimoto coefficient is 0.11.

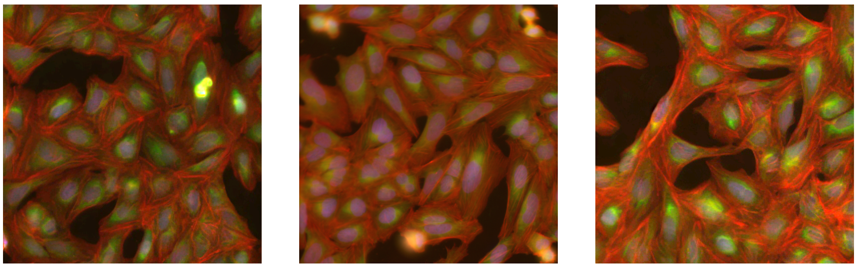

Supplementary Figure 3 Examples images of the neutral control treatments (dimethyl sulfoxide, DMSO).

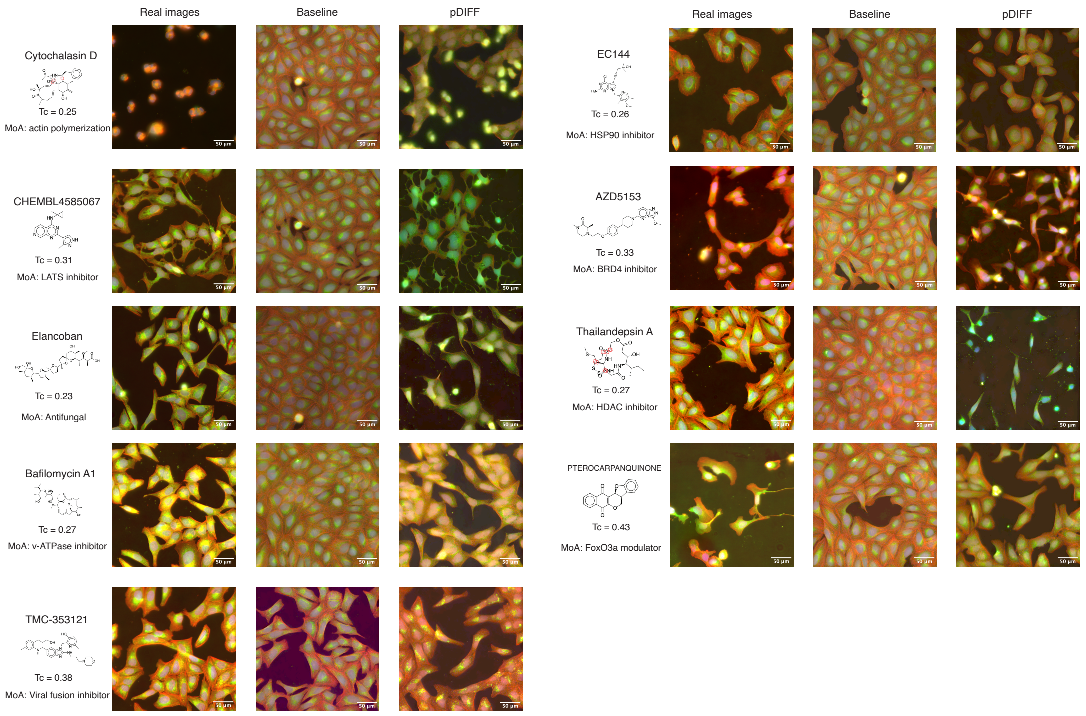

Supplementary Figure 4 Additional molecules in the realistic held-out set along with their corresponding real images (Nuclei, blue; mitochondria, green; red, cytoplasm) as well as images generated with diffusion models. We show images generated by a baseline diffusion model conditioned on chemical fingerprints (Baseline) as well as by pDIFF. For each molecule, we list the Tanimoto coefficient (Tc) to the nearest neighbor molecule in the training set as well as the associated mechanism of action (MoA).

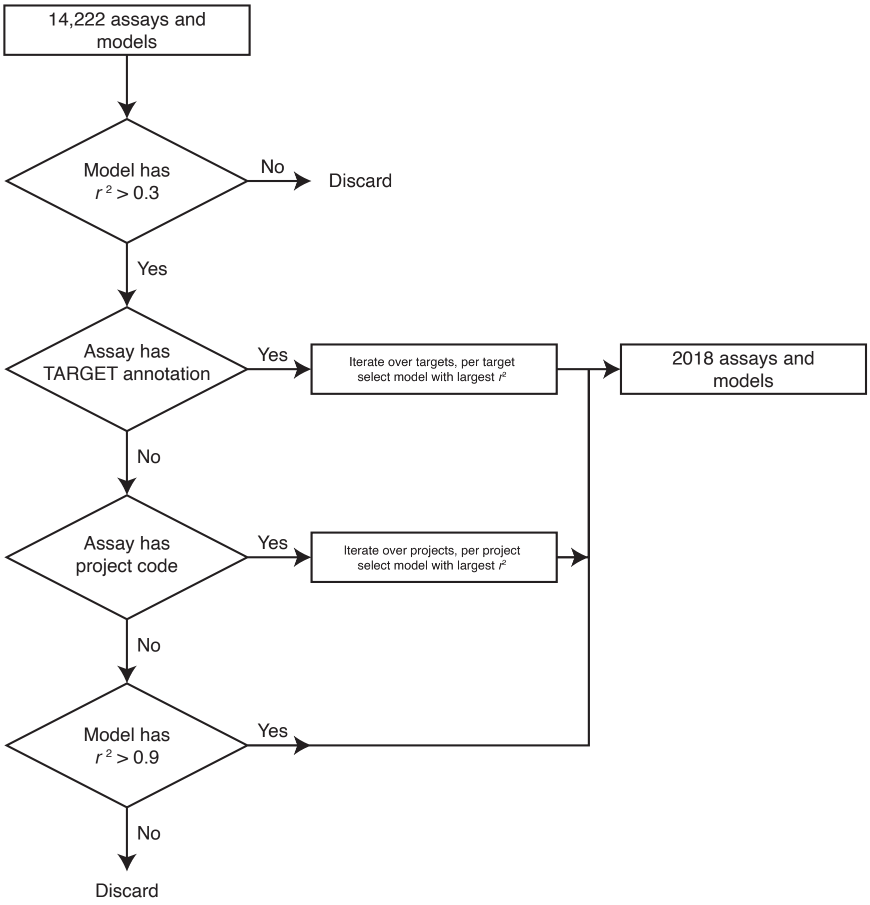

Supplementary Figure 5 Block diagram describing the process of narrowing down a 14222-D bioactivity profile to a 2018-D profile.

Supplementary Tables

Supplementary Table 1 Benjamini-Hochberg-adjusted p-values of two-sample Kolmogorov-Smirnov tests. Each test is carried out by comparing the distributions of percentage overlap values of two approaches.

	random	ECFP	pQSAR	Baseline	pDIFF
random		1.55e-03	3.45e-43	5.24e-07	1.85e-59
ECFP	1.55e-03		5.45e-28	3.97e-03	1.85e-59
pQSAR	3.45e-43	5.45e-28		9.19e-29	2.05e-13
Baseline diffusion model	5.24e-07	3.97e-03	9.19e-29		1.85e-59
pDIFF	1.85e-59	1.85e-59	2.05e-13	1.85e-59	

Supplementary Table 2 Training parameters for pDIFF.

Parameter	Value
batch size	6
gradient accumulation steps	10
GPUs	4x Nvidia A100 40GB
effective batch size	240
lr	1e-4
max grad norm	.1
training steps	30,000
training schedule	constant, 1000 step warmup
noise offset	.2
SNR gamma	5.0
optimizer	AdamW
adam beta1, beta2	.9, .999
adam weight decay	1e-2
adam epsilon	1e-8

Supplementary Table 3 Results for held-out images from training compounds. Values given are Spearman correlation coefficients between compound-aggregated average values for each image feature. The first row shows the correlation values between training images and held-out images.

Configuration	Coverage	Cell Count	Cell Size
Real images upper bound	.62	.53	.44
Baseline diffusion model	.41	.36	.15
pDIFF	.59	.46	.40
